# Management of Peripheral and Truncal Venous Injuries

**DOI:** 10.3389/fsurg.2017.00046

**Published:** 2017-08-24

**Authors:** Triantafillos G. Giannakopoulos, Efthymios D. Avgerinos

**Affiliations:** ^1^Vascular Surgery Department, Athens Naval & Veterans Hospital, Athens, Greece; ^2^Division of Vascular Surgery, University of Pittsburgh Medical Center, Pittsburgh, PA, United States

**Keywords:** venous injuries, endovascular, vena cava, inferior, thoracic veins, abdominal veins

## Abstract

Civilian injuries are increasing according to the World Health Organization, and this is attributed mainly to road traffic accidents and urban interpersonal violence. Vascular injuries are common in these scenarios and are associated with high morbidity and mortality rates. Associated peripheral venous trauma is less likely to lead to death and controversy remains whether ligation or repair should be the primary approach. Conversely, non-compressible truncal venous insult can be lethal due to exsanguination, thus a high index of suspicion is crucial. Operative management is demanding with fair results but recent endovascular adjuncts demonstrate promising results and seem to be the way forward for these serious conditions.

## Introduction

Over the past 50 years, additional advances in managing vascular trauma have been made in both civilian and military practices. These have included experiences with endovascular procedures, particularly over the past decade, transferring civilian experience to the management of battlefield (1). Amidst advances in graft materials and imaging seen during the 1950s and beyond, civilian experience with vascular trauma has developed rapidly.

Compared to arterial injury, venous trauma is related to decreased morbidity and mortality. Venous injury is less likely to lead to death especially when a *peripheral* vein is involved and the most likely result would be thrombosis of the affected vessel. Trauma-related venous thrombosis management can be difficult because hemorrhagic risk in the setting of concomitant injuries limits the use of systemic anticoagulation and may require a vena cava filter (2). *Truncal* vein injury, on the other hand, can cause life-threatening exsanguination and should be readily recognized and managed. Hemorrhage can be originating from either extremity or torso vessels, a distinction of significant clinical importance. Extremity hemorrhage is generally *compressible*, meaning those vessels can be accessible to immediate control with manual pressure or tourniquet application. This is in contrast to torso hemorrhage, which is usually *non-compressible* meaning vessels that cannot be controlled with direct pressure. Although extremity hemorrhage is a more common injury in trauma practice, non-compressible torso hemorrhage (NCTH) is accompanied by greater mortality (1, 3).

## Epidemiology

Vascular injuries in civilian life have increased in the past decade. This is due to more automobile accidents, the increase of gunshot and stab wounds, and the rising use of therapeutic and diagnostic techniques involving the cannulation of veins—leading to iatrogenic trauma. Injuries account for 9% of the world’s deaths, nearly 1.7 times the number of fatalities that result from HIV/AIDS, tuberculosis, and malaria combined (4). Injuries claimed nearly five million lives in 2015. More than a quarter (27%) of these deaths was due to road traffic injuries. Low-income countries had the highest mortality rate due to road traffic injuries with 28.5 deaths per 100,000 population—the global rate was 18.3 (5).

City populations can have high rates of interpersonal violence. However, there is considerable regional variation in violence rates. South Africa has an intentional homicide rate of 33.9 per 100,000, whereas the United States figure is 4.8 per 100,000 and the UK figure is 1.7 per 10,000 (6–8). The majority of vascular trauma in the city was carried by young men (86% male, average age 30 years), 90% of whom had been injured by firearms (gunshot wound 51.5%; shotgun injury 6.8%) or knives (31.1%) (9). The wound pattern in the civilian setting does not follow that seen in wartime. Torso and neck injuries account for two-thirds of all injuries treated, while lower extremity injuries comprise only a fifth (1, 10). Civilian injury hemorrhage is present in 15–25% of admissions vs 10% reported during combat (1, 11, 12). Civilian studies demonstrate that NCTH accounts for 60–70% of mortality following otherwise survivable injuries clearly emphasize the lethality of this injury pattern (3, 13). Studies on those killed in war action have shown that of deaths occurring by otherwise survivable injuries (10), 80% are a result of bleeding from disruption of vessels within the torso (1, 11).

## Peripheral Venous Injuries

The optimal management of *upper extremity* venous injury remains controversial. Ligation of upper extremity veins can be performed with low morbidity in austere conditions or when another injury takes precedence. Quan et al. (14) reviewed 103 combat venous injuries and confirmed that the majority of patients (63%) were treated with ligation without significant difference in postoperative thromboembolic complications compared to the repaired vein group. Limited civilian published experience (15, 16) has shown that more distal repaired veins tended to thrombose early without effect on morbidity while military reports note that when venous repair was undertaken, thromboembolic complications did not increase compared to ligation (17). Proponents of primary repair (18–20) argue that venous outflow restoration is more important in the proximal arm where large veins drain the majority of the limb axial outflow and when concomitant extensive soft tissue damage is likely to compromise venous collateral outflow.

**Figure 1:**
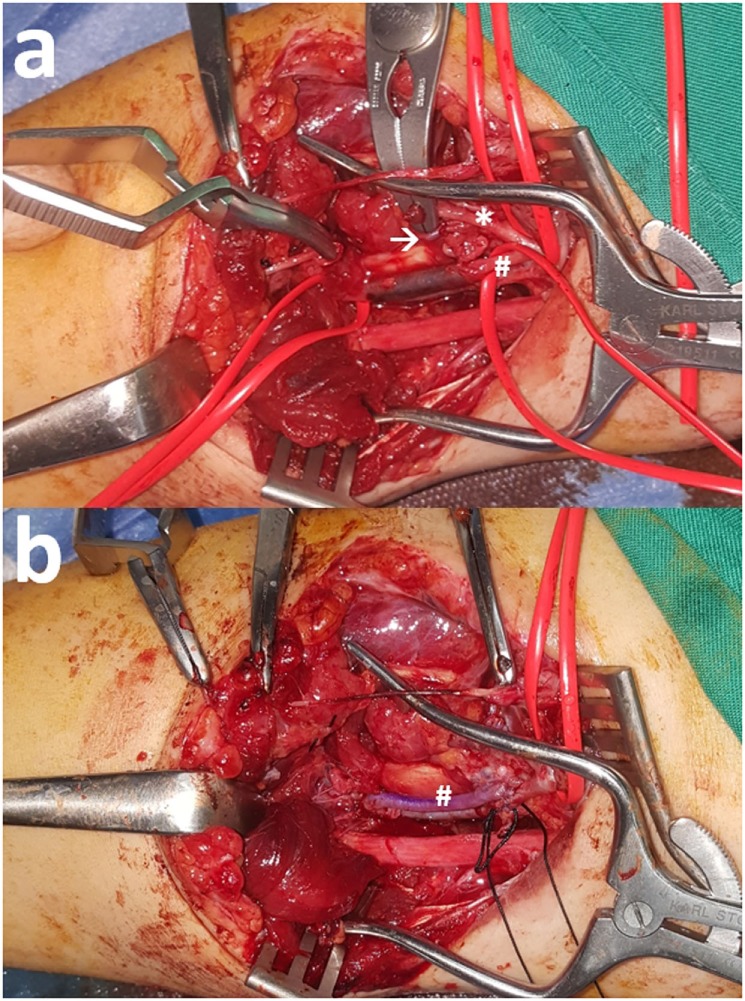
**(A)** Radial artery (*) transection along with ulnar artery (#) thrombosis after a road traffic accident. One satellite vein (arrow) is also transected but the other is intact. **(B)** Reconstruction of the ulnar artery (#) with interposition reversed vein graft and ligation of the rest transected structures was enough for full recovery.

**Figure 2:**
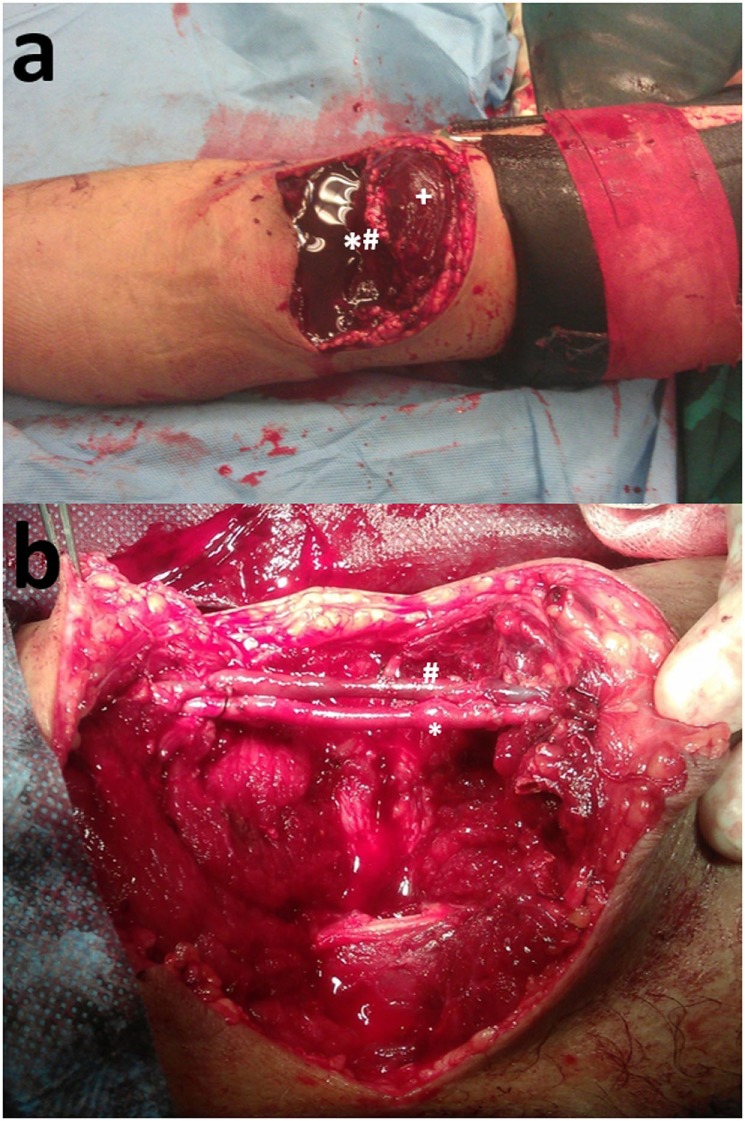
**(A)** Biceps (+) and brachial neurovascular bundle (*#) transection. **(B)** Reconstruction of the brachial artery (*) and vein (#) with interposition reversed vein graft.

Concurrent arterial compromise is the norm in cases of upper arm vein injury because of the tight anatomic vascular bundle formation. In the case of a 33-year-old female who presented with a traffic accident-induced radial artery transection, ulnar artery thrombosis, and transection of the one of its two satellite veins (Figure 1A), a vein interposition arterial reconstruction for the ulnar artery and ligation of the transected radial artery and neighboring veins were enough for a satisfactory recovery (Figure 1B). On another occasion, though, for a complete biceps and neurovascular bundle transection (Figure 2A), reconstruction was undertaken for both brachial artery and vein using saphenous vein (Figure 2B). Iatrogenic venous injury in upper extremity is an important attribute of vessel access procedures that can lead to deep vein thrombosis with significant morbidity. The extensive use of peripherally inserted central catheters has been linked with increased risk of upper arm deep venous thrombosis in cancer patients (21).

Regarding *lower extremity* venous injury, ligation is better tolerated and more common than arterial ligation. If the injuries are repairable and the patient is stable, reconstruction of popliteal, superficial, common femoral, and iliac vein injuries should be given serious consideration to reduce acute venous hypertension and longer term morbidity. Maintaining venous patency and outflow is especially important in these watershed or “gatekeeper” veins. For these injuries, military experience has shown that this selective repair strategy was effective with high patency and low thromboembolic events rate (14).

Techniques used for venous injury repair depend on the vein wall defect and vary from lateral venorrhaphy, end-to-end anastomosis, patch venoplasty, and interposition graft using autologous vein or prosthetic conduit. “The jury is still out” whether ligation or reconstruction should be the method of choice for these injuries and routine ligation proponents (19, 22) claim that venous stasis is mitigated by venous collaterals. On the other hand, selective repair supporters report acceptable patency results (14, 23, 24) when certain criteria are met. For those proximal veins that a decision has been made to reconstruct, a temporary vascular shunt may provide an interval option for more proximal vein injuries but use for more than a few hours will need to be aided by systemic heparin. Their use has been reported (23) to facilitate stabilization of orthopedic fractures before a plan for vascular repair is set and to decrease limb loss rates (25).

## Truncal Venous Injuries

Non-compressible torso hemorrhage is linked to increased mortality as vascular injuries cannot be readily compressed for control. Truncal veins are susceptible to this kind of injury, and one has to bear a high index of suspicion when treating a hypovolemic high-energy trauma patient (26) to the *thorax* or abdomen.

### Chest Vein Injuries

Penetrating injury to the superior vena cava, to the thoracic course of the inferior vena cava (IVC), and the right atrium has the potential to seriously compromise hemodynamic stability both by exsanguinations and by drastic reduction of the right atrium venous return. If the patient reaches the hospital alive, a median sternotomy reveals a defect that can be temporarily controlled by digital pressure, skin stapler (27–29), satinsky vascular clamp, row of Allis clamps, Foley balloon catheter (30) and crossed mattress sutures for bigger defects. In case of uncontrollable bleeding, caval inflow occlusion can help the surgeon achieve hemostasis for a minute or two due to induced severe hypotension and bradycardia. Before employment of cardiopulmonary bypass (CPB) circuitry, bolus 3 mg adenosine-induced short asystole (31, 32) has been used to achieve a quick suture repair. When control is impossible, the patient will need to be placed on CPB with the inferior cannula placed in the abdominal IVC *via* the femoral vein and a balloon catheter occluding the IVC beyond the injury. Air embolism is something to watch for just prior to major thoracic vein repair completion but can also take place after blunt trauma (33).

Crossover left *innominate vein* injury is managed by a median sternotomy and should be repaired if the patient is stable. If necessary, it can be ligated though and the left upper limb put in stockingette and monitored for superficial volar compartment pressure rise [>35 mm requires fasciotomies (34)]. Endovascular repair has been reported with encouraging results (35). *Subclavian vein* traumatic injury can lead to devastating exsanguination due to communication with the pleural cavity and should be promptly controlled by a high anterolateral thoracotomy at the third or fourth intercostal space and adequate packing of the pleural space together with supraclavicular fossa pressure. If repair is dangerously cumbersome, it is better ligated and the arm monitored for signs of venous hypertension. *Axillary, azygos* (36), *and pulmonary vein* injuries are highly lethal and even less frequent targets of injury that recent imaging advances has provided insight into their underestimated incidence (37).

### Abdominal Vein Injuries

Trauma injury to the IVC, portal (PV), and mesenteric venous (SMV) systems is not common and is accompanied by high mortality. Despite advances in care, the mortality associated with these venous injuries has not changed in the last 30 years and literature reports death rates of 50–70% for injuries to the SMV, the PV, and IVC (38–40). With IVC injuries alone, 30–50% of patients will fail to reach hospital alive (41). High mortality has been attributed to difficulty in operatively accessing the structures as well as hemorrhage from a high-flow, low-pressure system (39, 42).

Neighboring anatomy protects these vessels and, according to Asensio et al. (39), there coexist 2–4 associated organ injuries for every visceral vessel damaged. Penetrating trauma is the dominant (95%) type of injury to these structures (39, 43). The IVC is the most frequently damaged and requires complex decision-making. Therapy for this triad of injuries is mainly operative and prompt identification and surgical competence are prerequisites for a good outcome.

The overall incidence of *IVC injury* ranges from 0.5 to 5% of penetrating injuries and 0.6–1% of blunt trauma (44). Approximately 30–50% of patients will die before reaching the hospital (41, 44). Of the patients who survive to the hospital, 20–57% will not survive to discharge (41). As the IVC is a low-pressure retroperitoneal structure, bleeding is initially contained. Patients presenting stable with contained hematomas are candidates for non-operative management (45). Vigorous intravenous fluid resuscitation—especially through lower extremity access sites—must be avoided to reduce chances of tamponade release. Importantly, obvious signs of deterioration indicate failure of the current course of management and the need for surgical exploration.

Injuries to the infrarenal IVC have the best survival due to the ease of access and tolerance to ligation. The suprarenal IVC is relatively accessible but is more closely associated with sensitive neighboring structures while suprarenal ligation is poorly tolerated (43). Injury to the retrohepatic IVC almost and always includes damage to the liver parenchyma, and this allows free bleeding into the peritoneal cavity. Exposure is difficult and survival is low (46). Finally, mortality from injuries in the suprahepatic IVC approaches 100% due to difficulty gaining control in this region. When the injury is identified preoperatively, endovascular techniques will likely provide better salvage than open approaches. The use of endografts has been reported for treating retrohepatic IVC in conjunction with laparotomy (47, 48), with adjunctive Pringle maneuver/packing and as a primary intervention alone (49) or combined with fenestrations for hepatic vein drainage (50). Such a strategy could be beneficial in cases of iatrogenic caval avulsions are anticipated in “hostile” abdomen elective surgery or in cases of suspected caval invasion by malignancy. Still, controversy exists regarding anticoagulation to prevent thromboembolic events (50, 51).

In the presence of a retroperitoneal hematoma, the IVC is approached from the right. Specifically, left visceral rotation followed by an extensive Kocher maneuver is performed. Proximal and distal control of the IVC is advisable but is not always possible. If active hemorrhage is encountered, direct pressure on the area of injury should be applied. Then control is achieved by starting proximal and distal to this region and moving toward the defect. Control of the retrohepatic and suprahepatic portions of the IVC is particularly difficult to achieve (52) but retraction of the liver upward will allow access to the most proximal portion of the infrahepatic IVC. Complete mobilization of the liver by division of the suspensory ligaments will provide some mobility to access the retrohepatic portion of the cava but removes the possibility of tamponade by the organ. Access to the suprahepatic IVC will always require division of the diaphragm for adequate exposure and a sternotomy may be due for proximal control of such injuries (53). Percutaneous approaches that involve use of compliant endovascular balloons for inflow and outflow occlusion may be sought to address injuries to this portion of the IVC.

For penetrating injury, the common approach is to apply sponge sticks above and below the wound for proximal and distal control (41, 43), but this may widen the injury or create a new one. Direct pressure on the injury is better starting with one’s fingers. In the case of linear injuries, the vein edges may be grasped with Judd-Allis clamps and closed either with a Satinsky clamp or sutures. A simple stitch placed at the proximal and distal extent of the laceration, with gentle upward traction, will elevate and collapse the laceration and allow control and exposure for primary suture closure (54). Another consideration in repair of the IVC is the use of a larger non-cutting needle that can be visualized in the presence of considerable amounts of blood. The combination of an anterior and posterior caval laceration can be managed easier in the infrarenal cava by slight rotation. However, proximal IVC cannot rotate due to tributaries so that the posterior defect must be repaired through an extension of the anterior one. Finally, in cases of large defects, interposition grafting should be considered taking into account the considerable time needed for this cumbersome reconstruction.

Hemorrhage control presents unique challenges in the case of blunt retrohepatic and suprahepatic IVC injuries. Direct pressure on the liver parenchyma to reapproximate tissues and direct pressure posteriorly along with a Pringle maneuver should be utilized if the parenchyma is bleeding. When hematoma is identified behind the hepatic ligaments, division of the ligaments should be avoided (55). In such cases, endovascular single balloon control, caval isolation using two balloons (56), or caval stent-grafting (57) can solve the problem while atriocaval shunts are likely to become obsolete in the endovascular era (58–60). Open liver total isolation requires sternotomy or a right thoracoabdominal incision plus a Pringle maneuver and is rarely tolerated by the patient while the ultimate step involves supraceliac aortic clamping (58, 59). The use of circulatory arrest (52, 53, 61), veno-venous bypass, hypothermia, and liver autotransplantation (62) require demanding equipment, surgical team experience, and are time consuming, thus only marginally improving outcomes.

Ligation of the infrarenal IVC, iliac veins, and left renal vein are tolerated fairly well. Conversely, ligation of the PV and the SMV are poorly tolerated and while ligation of the right renal vein often results in kidney loss (63). Sullivan et al. reported that over a 13-year time period, 43% of patients underwent ligation and had a 59% overall mortality rate—compared to 21% of the repair group. The major morbidity of infrarenal IVC ligation is lower extremities swelling that is potentially severe enough to cause acute compartment syndrome. However, fasciotomy is not recommended as a routine prophylactic measure following IVC ligation (54, 64).

#### Portal Vein Injury

Portal vein injury occurs in as low as 0.1% of all traumatic injuries (65). However, associated morbidity and mortality is high and only 20% of patients with two portal triad structures injured survived in a series of 99 patients. Interestingly, 85% of intraoperative deaths occurred in patients with portal vein injury (66). The portal vein is accessed from the right with a wide Kocher maneuver plus a selective division of the head of the pancreas. The Pringle maneuver can help control bleeding but, in dire circumstances, the celiac artery needs to be clamped too (65). Repair is the way to go if patient status allows for the time needed for either primary suturing or interposition grafting with great saphenous vein. However, ligation is sometimes the only option and it has been shown that patients do better when ligation takes place prior to cardiovascular collapse (67, 68). Compared to IVC ligation, PV ligation is not as well tolerated and is accompanied by severe hypotension and intestinal edema that can further complicate recovery (40).

#### Superior Mesenteric Vein Injury

Superior mesenteric vein injury is as rare as PV injury. In the majority of cases, penetration is the mechanism of injury rather than blunt one caused by mesentery traction. Superior mesenteric artery is very often found to be injured also as they lie in close proximity at the base of the mesentery. Associated mortality is reported to be 50–71% depending on the number of concomitant solid organ and vascular injuries (38). When injury is distal to the pancreatic border, exposure is straightforward and repair is conducted in the usual manner. In case the laceration is closer to the pancreas, ascending colon and Kocher maneuver are due to allow for access to splenic-SMV confluence (39, 69). Ligation of the SMV when repair is not deemed safe or possible can be undertaken as long as it is performed before cardiovascular collapse. Reports of similar mortality rates (70) between repair and ligation patients support this notion, but ligation should always be followed by a second look laparotomy to allow for early identification of bowel ischemia secondary to venous hypertension.

## Conclusion

Civilian venous injury is rising mainly due to road traffic accidents and violent conflicts. Current literature suggests that traumatic peripheral vein injuries are not associated with increased mortality and should be repaired if allowed by the general status of the patient and concurrent comorbidities, especially when “gatekeeper” veins are involved. When needed, however, primary ligation is reported to be adequately tolerated. Truncal venous injuries, on the other side, carry significant mortality by exsanguination as they are not readily accessible to control by pressure. The increasing experience with endovascular techniques in elective vascular surgery provides exciting opportunities for applications in the management of vascular trauma. Case reports of such use are increasing in frequency, but controversy remains.

## Author Contributions

TG: conception, writing, editing. EA: conception and editing.

## Conflict of Interest Statement

The authors declare that the research was conducted in the absence of any commercial or financial relationships that could be construed as a potential conflict of interest.
